# An Ethnographic Exploration of Social‐Ecological Influences on Physical Activity in Care Homes for Older People

**DOI:** 10.1111/hex.70664

**Published:** 2026-04-10

**Authors:** Gavin Wylie, Thilo Kroll, Miles D. Witham, Jacqui Morris

**Affiliations:** ^1^ School of Health Sciences University of Dundee Dundee UK; ^2^ School of Nursing Midwifery and Health Systems University College Dublin Dublin Ireland; ^3^ Biomedical Research Centre Newcastle University Newcastle UK

**Keywords:** care home, older people, physical activity, social‐ecological model

## Abstract

**Background:**

Robust evidence confirms that physical activity (PA) levels among older people who live in care homes are considerably lower than those who live in their own homes. Low PA in care homes may reduce independence, function, and quality of life.

**Objective:**

To explore social ecological factors that influence PA facilitation in care homes.

**Methods:**

Focussed ethnographic study informed by social ecological models in five care homes for older people comprising 54 h of non‐participant observation and 15 in‐depth interviews with care workers. Observations focused on key interactions between staff and residents, the daily routines of care homes, and the physical environment. Interviews were informed by observations and sought explanations for observed behaviour. Data were subjected to reflexive thematic analysis.

**Results:**

The findings show how care workers' perceived roles, identity, and sense of purpose influenced how (or if) PA was facilitated. The study identified blurred role boundaries between formal and informal care practices in relation to PA. Blurred role boundaries led to a continuum between formal and informal PA facilitation practices that were mediated by intrapersonal, interpersonal, organisational and physical environmental factors. Formal roles in PA promotion were defined by their explicit inclusion in job descriptions, for example, staff employed as activity coordinators, and were highly demarcated leading to limited interprofessional collaboration among care workers regarding PA. Conversely, informal roles reflected how recognising and creating incidental opportunities to promote PA occurred for all staff as they enacted their roles during day‐to‐day work. Social spaces in the care home physical environment acted as destinations that encouraged walking and incidental PA, especially when supported by staff creativity and encouragement.

**Conclusions:**

These findings provide evidence to support the transformation of care worker roles in a way that emphasises PA facilitation as a key part of the role.

**Patient or Public Contribution:**

Care home staff, residents, and family members contributed to the design of the observation topic guide and the practical procedures to implement the observation element of the ethnography. Member checking of the themes with two care home managers and three members of care home staff that were participants in the study was conducted to obtain feedback on the findings.

## 1 Introduction

The proportion of the global population > 65 years of age is projected to grow from 10 per cent in in 2022 to 16 per cent in 2050 [1]. Decreasing mortality [1] and increasing disability and dependency [2] means that care homes are, and will remain a critical element of long‐term care for older people. It is therefore essential that care homes adapt to ensure that they are fit for an ageing population. In this paper, care homes are defined as 24‐h residential care settings providing care and support for older adults (> 65 years) both with and without on‐site registered nursing staff [3]. Physical activity (PA) promotion in care homes is an important strategy for minimising disability, maximising function, and reducing the need for unplanned care [4]. It is therefore important to find ways to maximise PA in care homes.

Physical activity (PA) is defined as “any bodily movement produced by skeletal muscle that results in energy expenditure” [5]. Global PA levels among older people living in care homes are low, with evidence suggesting that residents spend 79% [6] to 92% [7] of daytime hours inactive. Low PA levels are associated with a high risk of pressure sores, muscular contractures, decreased cardiovascular function, urinary infections [8] and sarcopenia – the progressive loss of muscle mass and function [9]. The prevalence of sarcopenia is high amongst care home residents and contributes to physical frailty independent of clinical disease [10, 11]. Frailty can lead to a cycle of low function leading to low activity, which in turn leads to disability, increased sedentary behaviour and reduced PA levels [12, 13].

Despite overwhelming evidence on the benefits of physical activity for older adults, its integration into care home settings remains inconsistent and poorly understood worldwide [14]. While policies in several countries advocate for active ageing [1, 15, 16, 17, 18], the translation of these goals into daily care home practice is hindered by several factors. Physiological changes and multimorbidity associated with ageing limit older peoples' intrinsic capacity for PA [19, 20, 21]. Consequently, many care home residents also take multiple medications, the side effects of which may impact PA [22]. Additionally, factors related to the care home context also impact on residents' PA. First, many care homes prioritise safety and care routines over opportunities for movement, resulting in sedentary lifestyles that exacerbate frailty, cognitive decline, and dependence [23, 24]. Second, privileging PA in care homes is challenging since care homes may have little capacity to initiate PA promotion interventions that take up care workers' time [25]. Third, care home staff may not engage in time‐limited exercise interventions to increase PA because of low belief in their utility, insufficient training and support, workload, and high staff turnover [26]. Therefore, factors influencing PA in care homes occur at several levels including the individual resident level, staff perceptions, and the wider care home organisational and regulatory context. There is therefore a critical need for contextually focused research that explores how PA can be meaningfully and sustainably embedded into the lived experiences of care home residents.

Most previous qualitative research has used a limited set of methods ‐ largely interviews and focus groups ‐ and it is suggested that studies would benefit from the integration of spatial methods (such as qualitative observations) to allow for nuanced analysis of environmental factors [27]. It is also argued that qualitative observational methods allow an in‐depth appreciation of complex social contexts – seen in care homes for older people – as well as other aspects of the environment that participants are unlikely to reflect on explicitly in interviews [28]. Additionally, whilst previous qualitative studies have explored single‐level influences on PA in care homes, including organisational factors or staff views [29, 30, 31, 32, 33, 34, 35], no previous studies have adopted a comprehensive social‐ecological perspective using an ethnographic approach to observe how PA is enacted in care homes in real time to account for the range of factors together that are known to influence PA in care homes [36]. In this study, we take an ethnographic approach in five care homes for older people with the aim to explore the diverse social, organisational, and environmental influencing factors on PA in care homes.

## 2 Methods

### 2.1 Study Design

An ethnographic approach was adopted to address the need for detailed prospective research that investigates the ways in which complex social contexts and processes in care homes influence PA. Ethnography allows the study of individuals' behaviour within their own naturalistic setting ‐ that is, real world and real‐time observations, rather than those carried out under experimental conditions [37]. Specifically, a focussed ethnographic approach was adopted. Focussed ethnography is a form of ethnography that concentrates on a specific scope, timeframe, aims, and population sample, and is therefore suitable for applied health research and aligned with the aims of the present study [38]. The researcher conducting data collection (GW) adopted the role of non‐participant observer, and therefore did not engage in any care home activities [39].

Data collection extended over a 23‐month period between October 2016 and September 2018. The study adhered to criteria for reporting qualitative studies (COREQ) (Supporting Information S1: File 1) [40]. The researcher conducting the data collection (GW) was a male clinical academic podiatrist with a master's degree, 18 years of clinical experience, including working as a clinical podiatrist in care homes for older people and training and research experience in qualitative data generation and analysis. The data were collected as part of a PhD study.

### 2.2 Ethics and Consent

Ethical approval was sought and obtained from University of Dundee School Research Ethics Committee (reference number: 2016003_Wylie). The focus of all observations were care home staff and residents, and the ways in which staff interacted with residents during the conduct of routine tasks. No personal information was collected during observations. Given that observations took place in communal areas of the care homes, it was likely that family members, visitors, other professionals and any number of care home residents would be present at any one time. Therefore, it was not possible to predict and account for every conceivable situation that may occur, and to consent every individual that may or may not be present. In that context, the following procedures were taken.

Care home staff, residents and others who were likely to be observed (e.g., visiting professionals, friends and family members) were made aware of the study by way of notices placed in prominent areas in the care home and a short general study information sheet that was placed in a number of prominent points in the observation area, informing visitors to the area that observations are taking place. The senior carer or nurse in charge also informed visitors of the study. Visitors who could potentially be observed received a copy of the study information sheet, and verbal consent was sought. Those who did not agree were not observed. Observation occurred with note taking only, rather than with audio and/or video recording. This procedure was developed following consultation with the participants, and Dundee University Ethics Committee.

This approach is justifiable in four ways: (1) field notes were entirely anonymised and no participant was be identifiable; (2) previous ethnographic work in care homes undertaken by the first author adopted this approach and had full ethical approval [41]; (3) there was no risk to the participants, either in terms of physical harm, identification, or disclosure of personal information because only observations took place and no intervention occurred; and (4) we presented the protocol to participating care homes in order to determine feasibility of the conducting the observations – all contributed to the development of the protocol, and all agreed that the final protocol was feasible.

We sought informed consent from prospective participants to conduct interviews, since these could be scheduled and pre‐planned. To ascertain ability to provide informed consent the study used a teach‐back method to assess participants' understanding and to assess their capacity to decide about participation. Potential participants were provided with verbal and written information about the study using clear, accessible language. Reasonable adjustments were made where required, including allowing additional time, using simplified explanations, and providing large‐print or abbreviated information sheets.

To confirm understanding, participants were asked to explain back the study in their own words. They were specifically asked to describe the purpose of the study, what participation would involve, that participation was voluntary and could be withdrawn at any time without consequence, and how their data would be managed, including issues of confidentiality. Participants were considered to have capacity to provide informed consent if they were able to demonstrate understanding and use of the relevant information in reaching a decision. Where a potential participant was unable to adequately explain these key elements despite appropriate support and adjustments, they were considered unable to provide informed consent and were not recruited. In accordance with the legal framework governing research in Scotland, no consultee consent was sought because there is no legal equivalent to the consultee process in Scotland that is used in other parts of the UK (England, Wales, and Northern Ireland).

### 2.3 Reflexivity and Positionality

The researcher conducting the data collection (GW) was a male clinical academic podiatrist with a master's degree and 18 years of clinical experience, including working as a clinical podiatrist in care homes for older people. This background shaped his sensitivity to issues of function and mobility in care home residents and provided an awareness of the organisational pressures faced by care workers. This contextual familiarity facilitated rapport with staff and supported nuanced interpretation of staff–resident interactions. GW remained attentive to the potential influence of his background on data collection and analysis, actively using fieldnotes and the involvement of co‐authors from different disciplinary backgrounds (geriatrics (MW), rehabilitation (JM), and psychology (TK)) to interrogate assumptions and interpretive perspectives. GW has training and research experience in qualitative data generation and analysis. TK, JM, and MW all have experience in qualitative data generation and analysis and contributed to data analysis for this present study but did not have direct contact with the participating care homes, staff, or residents. Reflexivity was addressed throughout the study through regular team discussions, use of reflective field notes, and collaborative analysis. Preliminary interpretations were challenged within the multidisciplinary team to minimise individual bias and to ensure that findings were grounded in participants' accounts rather than researchers' prior expectations.

In terms of positionality, the research team brought a range of professional perspectives to this study. These perspecives informed the study design but also carried the potential to shape assumptions about care practices and organisational constraints relevant to PA. The team acknowledges that their professional positions, prior experience, training, and perspectives may have influenced data collection, interpretation, and the framing of findings.

### 2.4 Informing Theories

**Figure 1:**
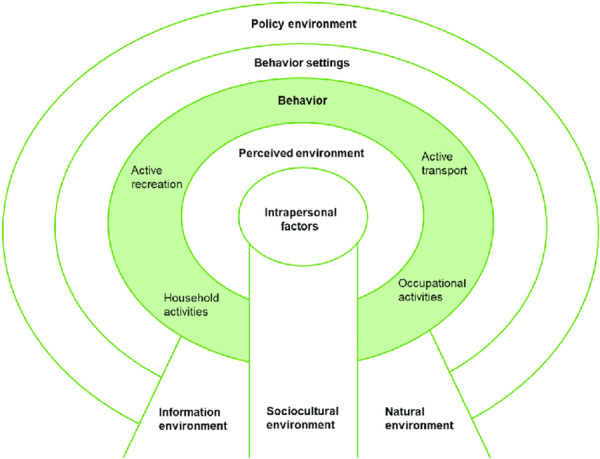
An ecological approach to active living communities [45].

Influencing factors for PA in care homes can be understood as a range of contextual factors that influence the uptake and maintenance of PA beyond intrinsic physiological capacity [36]. Consequently, social‐ecological models (SEMs) are useful to explain PA behaviour in older adults [42]. Social‐ecological models recognise that factors influencing behaviour adoption are multilevel, encompassing individual, social, environmental factors and the policy context [43]. Two social‐ecological models informed this study. First, the social‐ecological model by McLeroy et al. [44] was used because it includes concepts on individual and social‐environmental factors. However, McLeroy's model does not explicitly refer to the characteristics of the physical environment (e.g., building design and layout) that may promote or inhibit PA behaviour. Therefore, a social‐ecological model to inform the study was required that accounts for physical environmental characteristics. Consequently, this study was also informed by the Sallis SEM for active living communities which includes individual, organisational and physical environment concepts [45] (Figure 1).

There is ambiguity around the roles and boundaries of carers in care homes [46]. Therefore, this study was also informed by role theory which provides a conceptual framework that explains how individual staff behave and define their role in social situations [47]. Important concepts in role theory are ‘role ambiguity’ ‘role conflict’, and ‘role identity’. Role ambiguity indicates a lack of clarity on role expectations. Role conflict indicates role expectations as contradictory or mutually exclusive. Role identity describes individuals' interpretation of their role expectation in terms of norms, attitudes and behaviours required for a given role [47]. The SEMs informed both data acquisition and analysis whilst role theory informed data analysis and interpretation of the findings.

### 2.5 Study Setting

Seven care homes in total were initially approached to participate and five care homes for older people in Scotland in a single regional geographical area agreed to participate (Table 1). The participating care homes were all registered for older people living with dementia, physical disability or illness, and people with visual impairment. CHs 1,2, and 3 were residential homes with no registered nurse on duty, but nursing care was provided by regular visits from primary care nurses at least once per day. All CHs had at least one full time or part time activity coordinator present (Table 1). The characteristics of the resident population in the study care homes was in line with that of care home residents across the UK [48, 49].

**Table 1:** Characteristics of study care homes.

Care home ID	Location	Ownership	Resident capacity	Staffing	Physical environment/layout
CH1	Suburban	Local Government	28	Four care workers on duty during the day at one time. Part time activity coordinator	Bedrooms do not meet minimum room size standard set out in Scottish Care Inspectorate guidance [47] (12.5 to 13 m^2^ of usable floor space) and building design standards (floor covering and building materials) set out by regulator (Scottish Care Inspectorate) [47]. Main central lounge/dining area. Two operational units containing residents' rooms and its own lounge kitchen area. Garden.
CH2	Rural	Local Government	16	Two care workers on daily duty at any one time. Part time activity coordinator.	Does not meet minimum room size and building design standards set out in Scottish Care Inspectorate guidance [47]. Narrow corridors connecting communal lounge with residents’ rooms that do not minimum requirements set out by regulator [47] (≥ 1200 mm). One level; two units, each with own lounge. Large central lounge/dining room area. Enclosed garden area.
CH3	Town	Local Government	40	Five care workers on duty at any one time. Part Time activity coordinator	On one floor. Narrow corridors connecting communal lounge with residents’ rooms that do not minimum requirements set out by set out in Scottish Care Inspectorate guidance [47] (≥ 1200 mm).
CH4	Suburban	Sole proprietor – privately owned	54	Full time registered Nurse at all times; between 10–15 care workers on duty. Distinct teams for hotel services (four People), and activities (five people)	Large, converted house. The main building has wide corridors (> 1200 mm) and many different rooms serving different functions. No communal lounge. Modern extension with an open plan lounge area and residents’ rooms. Accessible enclosed garden area with pets.
CH5	Urban	One care home run by a private multiple operator	66	Two full time registered nurses on duty at all times. 15 care workers on duty at one time. One activity coordinator.	Modern purpose built (construction in the past five years). Large, bright wide corridors. Combined lounge/kitchen areas. Café style area at the centre of the home. Exceeds the building standards set out by regulator in all areas.

### 2.6 Care Home Identification and Sampling Criteria

Care homes were purposively sampled using a maximum variation approach based on care home characteristics known to influence how care is delivered: size (small resident capacity < 20, large resident capacity > 20), ownership (operator of multiple care homes, sole proprietor, or local authority), location (urban or rural), and design (modern purpose‐built, older purpose‐built, or converted older building) [43, 50]. Care homes were identified by contact with the statutory care home regulator in Scotland. Following identification, appointments for initial meetings were made. Emails containing study details were sent to care home managers ahead of each meeting to allow an informed discussion about the study.

### 2.7 Data Collection

#### 2.7.1 Observations

An observation topic guide (Supporting Informaiton S2: File 2) was developed that was guided by SEMs [45, 51] and an evidence synthesis [36]. The guide was pilot tested and refined in CH1. Following pilot testing, the *Physical Environment* section of the observation guide was adapted to capture residents lived experiences of the physical environment rather than simply describing its features. Observations focused on daily life routines, the ways in which staff interacted with residents, and how these interactions influenced PA. Observations were taken at three different time points throughout the day, and on different days of the week, including weekends: morning (0600 to 1000hrs), mid‐morning to mid‐afternoon (1100 to 1500hrs), and early evening (1600 to 2000hrs), including staff meetings and shift handovers. During observations the researcher was unobtrusively positioned in common areas in each of the care homes such as the living room, hallways, gardens, and offices. During observations, naturalistic interviews were conducted on an ad hoc basis, with the care home staff member interviewed as they went about their daily activities and asked to explain their perspective on things that arose spontaneously ‐ for example why they chose a particular course of action in a given situation. This emphasised their actions rather than relying only on what they said they believed they “would do”, or “had done”. These naturalistic interviews did not use a formal topic guide and were documented as part of the observation fieldnotes and a conversational format was adopted taking cues from observations that the researcher (GW) found interesting or surprising. Each observation session lasted between two and four hours. Written field‐notes were taken during observations, which were typed in full immediately after the observation session was completed.

#### 2.7.2 Interviews

Semi‐structured interviews with care workers were conducted to explore actions seen during observations [52], and non‐observable phenomena, such as beliefs. Consequently, the interview topic guide (Supporting Information S3: File 3) was derived from data collected during the observations. To participate in semi‐structured interviews, staff had to have been observed providing daily care for residents, and in permanent employment in the care home. We aimed to purposively sample a range of interviewees including care workers, senior carers, members of management teams, and activity coordinator staff, residents and family members. Interviews lasted between 45 and 60 min, were conducted in each of the care homes, digitally recorded, transcribed verbatim and anonymised.

#### 2.7.3 Sample Size and Saturation

Sampling continued until sufficient data had been collected that could populate the thematic categories derived from ongoing analysis to maximise the variety of data that supported each category (data adequacy) [53], via ongoing contemporaneous data collection and analysis. This approach enabled ongoing revision of the interview topic guide based on observations to facilitate understanding observed behaviour. Data collection continued until no new themes were generated and any further data collection resulted in saturation (analytical redundancy) – the point at which there were no more patterns derived from the data [54].

### 2.8 Data Analysis

Data were analysed using reflexive thematic analysis, following published guidance [55]. Data were first coded inductively before transposing these to informing theoretical frameworks to ensure that data were not ‘forced’ into predefined constructs. Open codes were developed that were descriptive of explicit, surface meanings of the data (semantic). Codes were also developed to identify underlying ideas, patterns, and assumptions (latent). Codes were then merged where similarities existed or modified to incorporate new data. Next, the codes were subjected to within‐ and cross‐case analysis (where each care home was a case) to identify areas of uniqueness and commonality, and to determine if the findings resonated beyond one care home. Thus, as well as inductively deriving themes from the data, the analysis was guided by concepts within SEMs and role theory. Trustworthiness was enhanced by member checking with a group of interview participants conducted near the end of the period of data analysis. The process involved returning the results of the analysis to check for resonance with their experience. The focus group was held at a location independent of the study care homes, where the key findings were explained back to the focus group members for verification and checking.

Since interviews were informed by observations, the analysis process led to the integration of interview and observational data across themes resulting in comparisons between interview and observational data being made, with the different sources of data bringing different perspectives on similar issues. Staff who were observed were subsequently interviewed to seek explanations for observed behaviour. Therefore, in presenting the analysis, we have largely drawn on interview quotes over observation fieldnote excerpts because the interview quotes offer readers efficient access to the critical analytic concepts. It is worth noting that in reporting the findings, where the term ‘activity’ is used, this refers to a range of activities that that aim to improve either residents' emotional wellbeing, cognitive status, or physical function [56]. In this context, ‘activity’ in its broadest sense in care homes is often seen as something distinct and not part of everyday routine. Therefore, it requires the support of additional staff whose job it is to co‐ordinate activities within the home (referred to in this study as ‘activity coordinators’ [57]). Physical activity can therefore be considered as one type of ‘activity’ [57]. Where physical activity is referred to specifically, this follows the definition “any bodily movement produced by skeletal muscle that results in energy expenditure” [5].

NVivo, Mac version 12 (QSR International, Australia) facilitated data management. Coding was conducted by GW and JM with the final themes derived from discussion between GW, JM, TK, and MW. To enhance trustworthiness, member checking with two care home managers and three members of care home staff that were participants in the study was conducted towards the end of the period of data analysis to obtain feedback on the findings. The process involved presenting the results, interpretations, and implications of the analysis to check for resonance with their experience [58]. The focus group was held at a location independent of the study care homes. Supporting Informaiton S4: File 4 shows the derivation of the themes from the coding process.

## 3 Results

### 3.1 Sample Characteristics

The observation dataset documented 54 h of observation across five care homes. Table 2 shows the characteristics of the interview participants. Fifteen semi‐structured interviews were completed with care workers (*n* = 6), senior grade carer workers (*n* = 4), care home managers (*n* = 2), and activity coordinators (*n* = 2).

**Table 2:** Characteristics of interview participants.

Participant	Care home	Years working in care home	Gender
Carer	CH2	13	Female
Carer	CH1	11	Female
Carer	CH2	3	Female
Carer	CH3	14	Female
Carer	CH3	15	Female
Carer	CH5	5	Female
Senior carer	CH4	9	Female
Senior carer	CH3	10	Female
Senior carer	CH3	14	Female
Senior carer	CH1	9	Female
Activity coordinator	CH5	5	Female
Activity coordinator	CH4	6	Female
Care home manager	CH4	12	Female
Care home manager	CH5	7	Female

### 3.2 Findings

At the intra‐personal, inter‐personal, and organisational levels, physical activity promotion existed on a continuum between formal and informal practice. Formal roles in PA promotion were defined by their explicit inclusion in job descriptions, for example, staff employed as activity coordinators, and were highly demarcated leading to limited interprofessional collaboration among care workers regarding PA. Conversely, informal roles reflected how recognising and creating incidental opportunities to promote PA occurred for all staff as they enacted their roles during day‐to‐day work.

There were also important influences at the physical environment level. Rooms in care homes emerged as ‘destinations’ for residents to walk to and explore. These social spaces facilitated PA because they were places that gave residents reasons to walk to, for example, dining areas, activity rooms, or gardens. They encouraged the formation of social groups of residents to form, facilitating social interaction and participation. The creation of destinations led to the emergence of walking routes for residents to use. Therefore, alongside intra‐personal, inter‐personal, and organisational levels, the physical environment shaped residents' use of the available care home space and influenced how PA was enacted. These data, explained in the themes below point to a spectrum of PA practice among carers, from work practices informed by formal organisational routines that constrained PA to more flexible and informal staff‐led practices that facilitated it. The member checking process confirmed the validity of the analysis.

### 3.3 Formal Organisational Culture in Physical Activity Promotion (Organisational Level of Social Ecological Model)

Formal organisational culture in PA promotion was embodied by the presence of specific staff with responsibility for PA facilitation, such as activity coordinators or external exercise providers. This arrangement for PA facilitation therefore relied upon a restricted subset of specialist staff, often excluding the care workers who were responsible for the daily care of residents.

Formal organisation of PA facilitation appeared to be most prevalent in CH4 and CH5. Both care homes were privately funded, which may partly explain the difference from the other study care homes. For example, in CH4, several activity coordinators formed a discrete team with a distinct role incorporating PA facilitation that was separate from carers, emphasising how professional boundaries determined that PA promotion was largely facilitated only by those staff who identified with the role:

… but because we've got the activities team as well…they are there just to be there for the residents…So, that (PA) wouldn't be a carer's role as such…but because we've got the five activities workers, that would be more their role to do that, rather than the usual care staff.

[Interview, manager, CH4]

In CH1, CH2, and CH3, activity coordination was an extended role taken on by a care worker – another form of formal organisational culture in PA promotion. This role was part‐time across multiple homes and led to the separation of the role of carer from PA facilitation:

I am approached by carer who mentions that the carer with an extended role as an activity coordinator is on holiday this week. I am told that other carers will attempt to take on that role in her absence, although there is no evidence of this today – carers are focused on essential care‐related tasks.

[Observation fieldnote, CH3]

Ostensibly, the identification of care workers with an extended role in PA was designed to increase resident's PA levels. However, there remained limited opportunity for PA for most residents because the peripatetic nature of the role in CH1, CH2, and CH3 led to time limitations; thus, staff had to be selective about the residents they worked with leading to a natural bias toward working with residents who were the most able:

The carer tells me that the two residents she is working with are relatively easy to work with and are already quite motivated and interested in increasing their [physical] activity levels. I am told that they have been successful.

[Observation fieldnote, CH2]

Care home practice that was informed by formal organisational culture often meant that on a day‐to‐day basis, PA was often not prioritised. This was underlined during observations in the nurse's office in CH4 during a carer handover meeting:

Two members of staff are going through each resident and sharing information. They discuss which residents require a bath today. A discussion is held about which residents need what kind of personal care today. They discuss medication, bowels, how much residents have eaten and other healthcare issues. How much residents have moved, or anything to do with PA is not discussed.

[Observation fieldnote, CH4]

Here, organisational meetings provided a ‘tool’ of formal organisational culture that reinforced the care worker role as one that does not include PA facilitation. Routine meetings, such as these handover meetings between shifts, informed and contributed to the deprioritisation of PA as a part of usual daily care.

### 3.4 Identity and Role Beliefs (Intra‐ and Inter‐Personal Levels of Social Ecological Model)

Many care workers in homes with PA facilitators did not believe that PA was an integral part of their job. Providing daily direct care was central to their identity, congruent with the formal organisation of PA facilitation:

… when the activities came into play, it was like, 'You're the activities… it's not my job, that's her job…you're the activities, you get on with it, that's not my role… it's the same as the domestics, “I'm not cleaning that, that's a domestic job.' … it's everybody's job…if I see something lying down, I'll go and pick it up.

[interview, manager, CH5]

Managers agreed that where PA facilitators were present, it was difficult to enable carers to see PA facilitation as part of their role:

Yeah, we've found it quite difficult to actually get staff to do that [facilitate PA], it's like it's not their job to facilitate it.

[Interview, Senior Carer, CH3]

Thus, perceptions of role identity appeared to affect the way care home staff integrated as a team and emphasised the challenges of fostering collaboration for PA across all care home staff. A collaborative and cohesive approach among all staff in terms of PA facilitation was not evident. As a result, the totality of care home work was achieved through the broader care home team conducting their work in seemingly discrete and segregated roles.

Staff articulated this personal belief of their role to their identity as a carer by describing a list of ordered and ‘approved’ tasks to ensure that residents' basic needs were met:

…my role in the morning would be ensuring the people that are still in bed are assisted or if they are able to do themselves, to ensure that clothing is appropriately on, hair and teeth and nails and that kind of thing. You then have the medication; so, we have 8 o'clock medication, then we do 9 o'clock medication and there might be meds in between and then it becomes breakfast time, lunch time, tea time, afternoon cups of tea, morning cups of tea, so again it's a full day of work the minute you hit the floor.

[Interview, Senior Carer, CH2]

In contrast to care workers who did not see PA facilitation as part of their role, there were also carers who blurred role boundaries between carer, domestic staff, and activity coordinator (CH1, CH2, and CH3). Here, there was broad support for informal cultures of PA facilitation. For example, senior staff valued the notion of carers working beyond the focus of essential care only:

I would like to move away from tasks like checking the rooms and things like that and spending more quality time. It's just not possible but we do spend as much time as we can with the client group.

[Interview, Senior Carer, CH3]

Some staff were comfortable finding ways to work differently. Working differently appeared to originate in a perceived shift from the notion of care homes as places that emphasised dependency to places that fostered independence:

You went and that was all you did, and you did everything for them, but nowadays, it's changed…for the better. Because we encourage them to go down the street. We encourage them to get their own paper, whereas we used to deliver the papers to their room. We now have a stand as you come in the door, and they have to go and get their paper. So, it's to try and encourage them, even little steps.

[Interview, Carer, CH2]

A key element of informal PA facilitation stemmed from carers who reported finding ways to incorporate PA into the daily routine:

I'm quite happy to let them fold the towels for me or when we put the towels out, I'll get one of them to follow me while we're doing it and we'll chat and then they'll put the towels in the bedroom for me. I'm trying to find ways, but I would make her go and get her cardigan herself if she could or go with her. And we'll have a chat. Let's go and have a chat and get your cardigan.

[Interview, Carer, CH3]

Staff also created conditions for informal PA facilitation driven by their interests, experiences and creativity. Consequently, how staff viewed themselves in terms of their identity, past experiences, creativity, and purpose influenced their actions in relation to PA:

I had a client that kept losing weight but then would pile it all back on… and I related to that completely for myself. So, she (resident) said the exercising was boring, which is how I feel about it as well. It is, it's boring. So, what I did is I made it fun for her. I made a treasure map, so her walk is internal round the home…each day she had to go and find something.

[Interview, Carer, CH3]

Here, the carer appeared to form an emotional and psychological connection with the resident through shared experiences, understanding, and beliefs. This experience led the carer to adopt a person‐centred approach to facilitate meaningful PA for residents. Discretion was used to respond to a situation in a way that was sensitive to residents' preferences, with the care worker feeling free to act on their judgement without deference to protocols or authority. This freedom for carers to act suggests that where informal roles existed, carers could be more responsive to residents' preferences with the potential to create PA opportunity.

Informal PA facilitation was also driven by staff exploiting incidental opportunities to facilitate PA. Staff acknowledged the need to promote and facilitate PA as they worked, thus exemplifying the informal role. Carers explained in practical terms how small, short bouts of PA (frequently characterised by staff using phrases such as ‘little bits’ or ‘little and often’) related to accomplishing elements of the daily routine were possible:

… instead of me saying I'm going to do this, I'll be like, oh, we'll go and get (resident's name) and we'll go and do that together.

[Interview, Carer, CH3]

### 3.5 Carers' Knowledge and Understanding of Physical Activity (*Intrapersonal Level of Social Ecological Model)*

Although formalised roles created specific behaviours among care home staff, many carers were aware of and knowledgeable about benefits of PA. Carers knowledge and beliefs about the role of PA for people in care homes were expressed as maintaining function or preventing deterioration and were critical for informal PA facilitation. For example, staff often recognised benefits of PA in terms of avoidance of specific physiological problems (e.g., pressure sores or joint contractures), which directly impacted the daily functioning of care home residents:

I think it's very important to have them moving around, because you can get pressure sores with sitting in the same spot too long. Their muscles can all seize up. You can end up stiff as a board if you're not moving anyway. So, even with our residents that are unable to walk and everything, they're totally off their feet, we do our best…

[Interview, carer, CH5]

The benefits of PA then became a resource that staff drew upon to encourage residents to be physically active:

I speak to them and let them know that if they don't use their legs, they're not going to work anymore, and they do need to use their legs as long as possible, because the last thing they want is not to be able to use their legs.

[Interview, Carer, CH5]

In addition to physical benefits, carers also explained how they felt that PA could facilitate residents' transition from living in their own home to living in a care home by improving low mood, fostering independence, and increasing residents' sense of personal competence:

She didn't want to be here, wanted to be at home…her mood was low and just with the physical activity improving gave her the confidence and now she smiles and she'll now just get up and walk by herself, where before she wouldn't even attempt to stand on her own, she would shout for members of staff, even for the toilet and little things, now she just gets up and goes.

[Interview, Carer, CH3]

### 3.6 Social Spaces as Destinations for Physical Activity (Perceived Environment Level of Social Ecological Model)

Social spaces included areas such as communal living rooms, corridors, office spaces and garden areas. Carers often adapted the physical environment in ways that facilitated PA. These adaptations were contingent on staff empowerment, flexibility, and creativity. This theme captures the notion of different physical areas of care homes emerging as social spaces and as ‘destinations’ for residents to walk to and explore.

Changes in room use made it possible for different physical spaces within the care home to be designated as destinations for walking to, thus providing opportunity for PA. How spaces in care homes were used largely depended on how care homes identified and used various spaces for different activities. Rooms arranged as social spaces often enabled PA:

…we encourage them to come up here for three o'clock tea rather than having it in their room. I set up with these old cups and saucers to make it look like just something different.

[Interview, Carer, CH2]

In CH4, formally organised social and physical activities such as exercise classes, group newspaper reading, reminisce, and quizzes occurred regularly and took place in a designated activity room. CH4 primarily consisted of several physical spaces, each with its designated use. In contrast, the internal layout of CH1, 2, and 3 meant that there were fewer physical ‘bricks and mortar’ spaces for residents to visit beyond bedrooms, hallways, and the communal lounge. Consequently, in these homes, the communal lounge was the focus of most daily life and where residents spent most of their time. Given the lack of different physical areas, residents in CH2 rarely interacted with other parts of the homes beyond either their own room or a communal lounge.

Social interaction between staff and residents about use of the physical environment influenced how and when residents used care home space. Encouragement to walk was particularly evident in CH2, where, in addition to the main communal lounge, there were two seldom‐used satellite lounges:

The wing sitting rooms *[referring to the satellite lounges]* are generally used for when family come to visit, we do encourage them to go up there, rather than sitting in front of everyone because it's not very personal to speak about anything.

[Interview, Carer, CH2]

Overall, the properties of the spaces within homes – which could influence meaningful activity – appeared to be insufficient to encourage residents to walk to and use these spaces. Instead, the room's location and intended purpose combined with encouragement from staff determined residents' physical movement into the area.

## 4 Discussion

**Figure 2:**
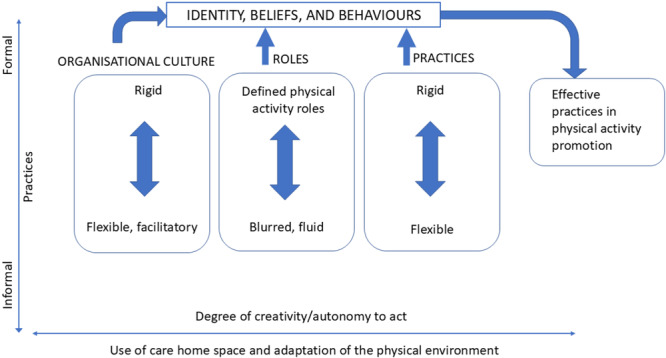
Conceptual diagram showing how organisational and individual factors influence how PA facilitation is enacted.

Factors across the levels of the SEMs influenced PA, with staff beliefs, staff perceptions, and physical environments interacting with one another to enable or constrain residents' opportunities for PA. An important contribution of this study is the identification of blurred role boundaries between formal and informal care practices in relation to PA, which were mediated by intrapersonal, interpersonal, organisational and physical environmental factors Figure 2 shows a conceptual diagram showing how organisational and individual factors influence how PA facilitation is enacted. The diagram depicts the study's empirically derived model, based on the findings from the ethnographic data. It operationalises and extends the informing SEMs by showing how levels of social‐ecological constructs interact in care homes to shape PA facilitation. Specifically, Figure 2 highlights how the interplay between organisational culture, the physical and social environment and individual role identity mediate staff behaviour and collectively determine whether PA is constrained or enabled.

Formal roles (such as activity coordinators) were demarcated, leading to limited interprofessional collaboration and siloed working among care home staff regarding PA. Consequently, PA was often perceived as the responsibility of specific staff. Conversely, in settings where PA facilitation was informally integrated into care routines, staff were more likely to embed PA in daily interactions and show flexibility in their role to adapt to fluctuating situations. Informal roles in PA promotion were driven by carers who worked across organisationally defined role boundaries drawing on autonomy, creativity, initiative, and personal connection with residents. This aligns with existing findings in studies set in other contexts that highlight the value of boundary blurring and flexible role interpretation in enabling adaptive and responsive care practices [59, 60]. Additionally, the findings from our study highlight an important tension: while formal organisational structures provide consistency of care, these structures may inadvertently constrain opportunities for staff to act autonomously or creatively. Informal facilitation of PA, although more flexible, depended heavily on individual staff members' motivation, confidence, and perceived autonomy ‐ often requiring staff to have the confidence to feel sufficiently empowered to act in ways that might be perceived as inconsistent with their role.

Care is often delivered in care homes for older people in a highly routinised way [61] which may be a barrier to PA in care homes [23, 24]. However, our study shows that care home staff were willing and capable of modifying and blurring role boundaries within care routines to facilitate PA. Importantly, this was contingent on staff seeing PA facilitation as an integral part of their role identity and linking the benefits of PA to functional ability and the avoidance of future problems. There is often ambiguity around the roles and boundaries of carers in care homes [46], and this ambiguity partly explains how perceptions of roles of carers in relation to PA facilitation were inconsistent across the study care homes. The role of identity and beliefs about the nature of work in care homes emerged as a critical intra‐ and interpersonal influence on how (or if) PA was facilitated. Staff who identified strongly with task‐oriented definitions of caregiving appeared to be less likely to view PA as part of their remit. In contrast, those who saw their role as supporting residents' independence and quality of life were more inclined to incorporate PA into routine tasks. Whilst previous research suggests that care home staff perceive safety as a factor that limits resident participation in PA [62], this was not a concern reported in the present study. This may reflect the characteristics of the participating homes, where staff were generally confident in residents' mobility profiles, familiar with their movement patterns, and accustomed to supporting low‐intensity, routine activity within predictable environments. It is also possible that the forms of PA observed – mainly short bouts of incidental walking – were viewed by staff as posing minimal risk. Nevertheless, evidence indicates that safety perceptions can influence staff behaviour [62]. Future studies should therefore examine how safety‐related decision‐making varies across homes with different resident profiles, staffing levels, and cultures of risk management.

Previous research has shown that around a third of care home residents report little or no interest in exercise programmes in care homes and that less than half of all residents participated in such programmes [42, 63]. This may be because many care home residents regard ‘exercise’ as inappropriate, often perceiving it as a strenuous activity only for younger people [42, 64]. This position risks inequality of opportunity: residents who may benefit the most from PA may not receive the opportunity to engage in any other form of PA. However, walking is often cited as a preferred form of PA in care homes because it is accessible and requires no equipment or specialised skills [65, 66]. The findings from our study suggest that staff creativity and empowerment combined with a physical environment that promotes walking (e.g., by creating walking destinations for residents) creates conditions that are conducive for PA.

Given the accessibility of walking as a form of PA for care home residents, it is important to consider wandering – defined as persistent walking amongst people with cognitive impairment, that care staff or relatives may find difficult to support [67] – in the context of the present study. Previous research has critiqued wandering as challenging behaviour to be managed rather than as a legitimate form of PA [68]. However, subsequent work has re‐conceptualised wandering behaviour as one that should be accepted and encouraged [67, 69]. This present study points to a need to encourage residents' naturally occurring behaviour in a way that is supported by the physical environment and care home policy, recognising that residents walk for reasons other than reaching destinations.

Care home staff were able to adapt the physical environment within existing infrastructure in ways they saw appropriate – for example setting up rooms a walking distance away from a communal lounge for socialising. Building layout therefore emerged as an important factor that shaped residents' PA. Homes with multiple communal areas, accessible corridors, or clear destinations created more natural opportunities for walking, whereas layouts centred around a single lounge tended to limit movement. However, the influence of layout depended on how staff used the space; in several homes, staff actively repurposed rooms or positioned activities in different areas to encourage residents to walk between them. This suggests that while environmental design can enable or restrict mobility, its benefits are realised when staff intentionally facilitate use of available spaces. These findings extend previous work suggesting that homelike environments, lighting, and sensory adaptations such as music may increase PA levels [43, 70]. Previous work also supports the role of the physical environment in assisting those with dementia to perform a range of everyday tasks, which may in turn promote PA. One study from Canada found that tailored individual environmental adaptations including providing equipment, labelling drawers and doors, alongside verbal cueing led to significant improvements in motor abilities [71]. Architectural features including smooth flooring, automatic doors, and walking loops are also reported to be beneficial [31]. Future care home design should therefore consider how spatial configuration can promote incidental PA as part of everyday routines. Creativity and autonomy were also central to care workers ability to adapt the physical environment, which was found to be important in PA facilitation in care homes during Covid‐19 lockdowns [72].

Our findings explain why role characteristics and definitions for care staff in relation to PA need to be clearly and explicitly defined. This could occur at the level of care home practices ‐ for example by embedding PA discussions into shift handovers and care planning to help bridge the gap between formal expectations and informal enactment. Care workers could integrate PA into everyday routines through small, incidental opportunities such as encouraging residents to walk to meals, collect items, or participate in light household tasks. To support and empower care staff in facilitating PA, senior carers and team leaders could play a key role in modelling and reinforcing flexible approaches to care that value PA. For example, this could be through embedding discussions of residents' movement and mobility into shift handovers and care planning meetings, ensuring PA becomes a standing topic alongside hygiene, nutrition, and medication. Care home managers should establish a culture where PA facilitation is seen as a shared responsibility. This may involve revising job descriptions to explicitly include PA promotion and recognising staff who innovate or adapt the environment to enable resident movement. Managers should also ensure the physical layout supports walking routes and accessible destinations, and that policies (such as open‐door access) encourage mobility rather than restrict it.

Change may also occur at the policy level. For example, inspection criteria could also play a role in reinforcing PA as an integral component of care, rather than a supplemental activity by incorporating observation of PA behaviours as part of inspection processes. It will also be important for those overseeing the admission of older people into care homes to discuss expectations of care practices in care homes with prospective residents and their relatives. These discussions should emphasise care homes as places where appropriate PA facilitation by all staff is usual practice. This work also informs the physical layout and design of new care homes ‐ the inclusion of multiple walking destinations may be useful; however, this needs to be objectively tested in future studies.

This study provides an important contribution in terms of exploring how PA is a product of interactions between individual, interpersonal, and the organisational and physical environment in care homes. Our ethnographic approach is novel in that it facilitated real time exploration of influencing factors at all levels of the SEMs simultaneously, allowing the qualitative interactions between the SEM levels to be explored. Crucially, the work presented here develops concepts for each SEM level that are important influencing factors for PA. These may also be targets for empirically derived hypotheses to develop and test interventions.

Some limitations to our study require comment. First, our study was conducted in five Scottish care homes, and findings may differ in settings with different regulatory environments. Nonetheless, our study recruited multiple care homes with diverse characteristics, incorporating a range of sizes, models of ownership, and a mix of rural and urban settings. Sampling therefore included a range of care home contexts offering rich, context‐sensitive insights that can inform practice. Additionally, the resident population was representative of the UK care home population [48, 49]. Although there was a lack of diversity among recruited care home staff, the sample is representative of care home staff in the area where the study was conducted (Scotland) rather than care home staff generally. There was, however, diversity in terms of seniority and experience. Second, data collection occurred pre‐COVID‐19, and subsequent changes in care practices since the pandemic may influence PA facilitation. Third, although residents were included in the observation data, no residents could be recruited for the interview phase of the ethnography. This was because of inability to provide informed consent following consulting with care home managers and care staff who acted as gatekeepers. Fourth, observations were limited to communal areas of the study homes, thus staff‐resident interactions in private rooms could not be observed. Fifth, although the data for this study were collected between 2016 and 2018, the analysis remains relevant because environmental and organisational, factors that influence PA levels have not significantly changed (care home design, staff/resident ratio, access to outdoor spaces) and although care home resident acuity increased substantially between 1992 and 2014, there has not been a substantial change since 2018 [73]. Finally, observations were limited to communal areas of the study homes, thus staff‐resident interactions in private rooms could not be observed.

## 5 Conclusion

Given the critical role of care homes as part of the care system for a subset of the population of older people, it is now more important than ever that novel, embedded and sustainable approaches to maintaining function in older people are implemented. These data provide evidence to support the transformation of care staff roles to emphasise PA facilitation as a key part of the role. In terms of future interventions and policy to increase PA in care homes, there should be a focus on widening roles and diffusing responsibilities of carers to ensure effective collaborative working for all care home staff related to PA facilitation. Future research should explore the long‐term sustainability of informal PA facilitation and test interventions that integrate the findings into care home routines.

## Author Contributions

**Gavin Wylie:** project administration, funding acquisition, conceptualisation, ethics approval, methodology, data collection, formal analysis, writing – original draft, review and editing. **Thilo Kroll:** supervision, conceptualisation, formal analysis, writing – original draft, review, and editing. **Miles D. Witham:** Funding acquisition, supervision, conceptualisation, formal analysis, writing – reviewing and editing. **Jacqui Morris:** supervision, conceptualisation, ethics approval, methodology, formal analysis, writing – oringal draft, review and editing.

## Ethics Statement

Ethical approval sought and obtained from University of Dundee School of Health Sciences Research Ethics Committee (reference: 2016003_Wylie).

## Conflicts of Interest

The authors declare no conflicts of interest.

## Supporting information


Supporting File 1

Supporting File 2

Supporting File 3

Supporting File 4

## Data Availability

The data that support the findings of this study are available on request from the corresponding author. The data are not publicly available due to privacy or ethical restrictions.
