# Supplementary material for: An Ethnographic Exploration of Social‐Ecological Influences on Physical Activity in Care Homes for Older People
Source: Health Expect. 2026 Apr 10;29(2):e70664. doi: 10.1111/hex.70664 (PMC13066766; doi:10.1111/hex.70664)
Supplement: Supplementary file 2 — Supporting File 2 [file HEX-29-e70664-s002.docx]

## Supplementary Material 2

## Observation topic guide

### Interpersonal factors

#### Visitor/staff/resident relationships

- What interactions occur between staff and residents that creates, constrains, or sustains opportunities for physical activity?
  - How do staff engage with residents?
- What role do visitors to the care home play in encouraging (or not) physical activity?
  - In what way are these roles enacted?

#### Decision making

- In what ways are decisions made regarding movement between residents and staff?
  - Who drives the action – Staff? Resident? Relative? Other?

#### Staff knowledge of residents

- In what ways are residents’ needs and preferences about physical activity and past experiences elicited with regards to physical activity?
  - How does staff appear to use their knowledge of residents’ preferences and past to motivate them to be more physically active?

#### Autonomy

- What are the ways in which residents are involved in decisions regarding their daily life (i.e.is autonomy fostered)?
- How is residents’ autonomy/preferences respected – choices about opportunities given or negotiated?

### Organisational factors

- Flexible/rigid staff routines/roles – In what ways does staff working routines appear to be task-oriented?
  - In terms of established care home routines, what facilitates, constrains or sustains physical activity?
- What tasks take away from physical activity encouragement?
- Number of staff on duty at any one time? What roles do they fulfil (e.g. domestic? caring?)

### Physical Environment

#### Observable physical features that are independent of situation/interaction

- Describe care home
  - Size (Number of rooms; % occupancy, staff : resident ratio)
  - Purpose built? Converted older property?
    - What are the features of the physical characteristics of the care home environment which may influence physical activity participation:
      - Prompts *(adapted from: Stark S, Hollingsworth HH, Morgan KA, Gray DB. Development of a measure of receptivity of the physical environment. Disabil Rehabil. 2007 Jan 30;29(2):123-37)*:
        - Short distance to travel from street/car park to building entrance?
        - Does the entrance to the care home contain stairs?
        - Are doorway thresholds low, and are there minimal gradients on any pathway?
        - Are internal and external door easily operated? Are they light enough to be opened with ease? Can they be opened one handed? Do they stay open long enough for a resident to pass through it? Automated? If automated, is the push button accessible?
        - Are doorways wide enough to accommodate users passing through whilst using a mobility device (e.g. walking stick/frame)
        - Is the care home located on a single level? If not, is there access to an accessible lift?
        - Toilets and bathrooms big enough to move around in whilst using mobility equipment?
        - Handles, sinks, paper towel dispensers easy to reach and manipulate?
        - Wide spaces to move through easily including enough room to turn around and change one’s path of travel?
        - Floor surfaces do not have inclines, bumps, or hills?
        - There is enough lighting for residents (is lighting level equivalent to outdoor daylight level (2)) to see where they are going and what they are doing?
        - All accessible features (doors, lifts, ramps) are in good working order?
        - There are accessible places to sit?
        - Are accessible paths well marked? Outdoor areas have signage located at accessible entrances?
        - There is signage directing residents towards accessible features?

#### How is the physical environment used?

- How do residents engage with their environment?
  - Prompts:
    - What is it like to move around the care home?
    - What environmental/physical features facilitates or constrains physical activity?
    - In what ways is the use of the care home space facilitated?

### Strategies

- What do staff do to encourage physical activity?
  - What staff behaviours facilitates, constrains or sustains physical activity?
- When are strategies used? Certain times of day?
- Who instigated the strategy?
  - How did the instigator of the strategy engage with the resident?
- Context of strategy – what was happening at the time strategy was instigated?
- Is there tailoring of strategies – if so, how?
  - Understanding preferences
  - Goal setting
  - Monitoring
  - Feedback
- Are strategies enacted policy? (Useful to do document analysis to start with in order to establish this?)
- At what levels do strategies and/or activities appear to operate
  - Individual staff member/visitors/external facilitators?
  - Organised approach from care home
  - Policy driven (if a written strategy, how is it articulated?)
- Strategy successful?
  - Did the resident move?
  - Long or short term increase in PA participation

## Indicative interview guide - staff

### Research Question 2:

1. *In terms of encouraging participation in physical activity, what are the beliefs, barriers, drivers and attitudes of:*
   1. *Care home staff (varying levels of seniority)?*
   2. *Care home residents and their social/familial network?*

### Individual/interpersonal factors

#### Background information

- Introductions, explain study, etc: I am a PhD researcher at Dundee University. Before we go on, I would like to briefly give you an overview of what this interview is about. Taking part in physical activity, such as walking, is good for health. However, we know that care home residents do not do much physical activity. To improve this, we need to understand why. One way to find out is to ask care home staff some questions about thoughts and experiences of physical activity among care home residents. Whatever experiences and views you have about physical activity in the care home, we would like to hear from you.
- Describe job
- Enjoy job?
- How long have you worked as a carer?
- How long have you worked here?

#### Meaning/understanding of PA

- Can you explain what do you think is meant by the term, ‘physical activity’?
- Might ask them what their thoughts are about PA, and what they do themselves, to give a background and context
- What are your views about promoting physical activity with care home residents?
  - How do you think encouraging residents to engage in physical activity fits into your role?
- What benefits do you think physical activity might bring to your residents?
- How important is promoting physical activity considered to be?

#### Influencing factors

- In what ways and by whom are you encouraged to support residents to be physically active?
- How/in what ways does your organisation encourage you to support residents to be physically active
  - Policy/rules
  - Training
  - Environment
  - External/internal facilitators
- What helps you to encourage residents to be physically active?
- Other than resident’s health status, what types of things prevent you from encouraging them to be physically active?
  - Is there anything concerns or worries you that might prevent you from encouraging residents from being physically active?
  - Can you give some examples?

#### Strategies

- Do you have any ideas about how care home residents could be more physically active?
  - Prompt - Any changes that could be made? Environment, social support, activities?
- Have you intentionally engaged residents’ in physical activities before?
  - How do you encourage residents to participate in physical activity?
  - Think about a recent example when you encouraged someone to be physically active
  - What led you to decide to do this?
  - How did it go?
  - Anything you might have done differently?
- Can you tell me how any visitors (e.g. relatives, volunteers, external staff, health care staff) encourage residents to be physically active? What helps/hinders?
- How does your organisation support staff to help residents to be more active?
  - Resources
  - Training
  - Policy etc
  - Other staff?
- What support do you think might help, that is not already in place?

#### Knowing residents

- Very generally, what sorts of things do you know about residents (in terms of their past, their preferences)
  - Can you describe how (or if) there are occasions when you use what you know about residents to engage them in physical activity?
  - How do you find out what might motivate a resident to be more active? How helpful do you think that information is?

#### Organisational factors

- Among staff, do you discuss increasing physical activity among your residents?

How do you think the care home organisation views facilitation of PA with residents?

How do you know that? How is that information conveyed to staff?

Are there any initiatives that promote physical activity in care homes? How are they viewed?

- Do you have any thoughts on their policies, or staff directives?

#### Environmental factors

- What sort of environment do you aim for here?
  - Prompt - e.g. homelike, any particular interior design features, such as lighting, sensory stimulus such as music?)
- What features of the care home help or hinder residents to be active
  - Prompts – building layout, distances residents need to go to fulfil activities of daily living, access to outdoor space, willingness to use outdoor space, are spaces within the care home accessible (e.g. doors easily opened, door wide enough)?
- What environmental/physical features facilitates or constrains physical activity?
- In what ways is the use of the care home space encouraged?
