# Supplementary material for: An Ethnographic Exploration of Social‐Ecological Influences on Physical Activity in Care Homes for Older People
Source: Health Expect. 2026 Apr 10;29(2):e70664. doi: 10.1111/hex.70664 (PMC13066766; doi:10.1111/hex.70664)
Supplement: Supplementary file 3 — Supporting File 3 [file HEX-29-e70664-s003.docx]

## Supplementary Material 3

## Interview topic guides

*(Note: Not all topics were relevant to all care homes, and this is indicated where appropriate)*

## Topic guide for care staff, except care home managers

#### Background information

Introductions, explain study, etc: I am a PhD researcher at Dundee University. Before we go on, I would like to briefly give you an overview of what this interview is about. Taking part in physical activity, such as walking, is good for health. However, we know that care home residents do not do much physical activity. To improve this, we need to understand why. One way to find out is to ask care home staff some questions about thoughts and experiences of physical activity among care home residents. Whatever experiences and views you have about physical activity in the care home, we would like to hear what you think.

To begin with, would just like to ask a bit about your job here.

- How long have you worked as a carer?
- How or why did you come into this line of work?
- Enjoy job?
- Could you describe to me typical day at work?

From what I have seen during my time here, and from what you have said - clearly, as a carer there are many tasks you need to do.

- Do you have the room to be flexible in what you do on a day-to-day basis? If so, can you give me an example?
- Do the tasks that you are required to do, interfere with more informal, less planned types of interactions with residents – for example just sitting down for a chat, or going for a walk with a resident? If so, how?
- If you had all the time in the world (relatively speaking!), how would you spend time with a resident?

As an observer, I see staff helping residents a lot – for example fetching things for them.

- Can I ask, why do you do this? *(Prompts: Do you do this because you feel it is instinctive to you to provide assistance? It is your job role to ‘help’ residents?)*

I’d like to ask about normal daily life for residents in the care home, and how your residents use the care home space available to them. I have already spent quite a bit of time in the care home, simply observing residents’ day-to-day lives. During the observations that I have been doing, one of the things I have noticed is that many of the residents seem to prefer to spend time in their rooms.

- Why do you think this might be?
- Do you see trying to get them out of their rooms as part of the daily care for such residents? How might you get residents to leave their rooms?
- As a carer, how do you balance allowing residents the choice of staying in their room with encouraging them to get out?

One of the other things I have noticed during my observations is that, when residents are out of their rooms, residents here still only use a small amount of the available care home space. For example, whilst there are small kitchen/communal areas in each of the wings (Care home 2 only), I noticed that residents rarely use these.

- Why do you think this might be?
- Would you encourage resident to use more of the care home space that is available to them?

*(All care homes except Care home 4)* From my time here, I have noticed that mealtimes can be quite a focus for activity. One of the things I have noticed is that many of the residents are brought through to the dining area very early for a meal, and may sit waiting for a long period of time before food it served?

- Can you tell me why this might be?

*(Care home 3 staff)* One of the things I have noticed is that the layout of this care home is quite confusing (to me!). I say this because there are lots of corridors, but they all look the same, and initially I found it quite difficult to locate where I was exactly.

- How do you think this might affect they way residents move around the care home?

All residents are different, and have different abilities, but what do you believe that care home residents in general, are capable of (think about how much they can move, what decisions they can make for themselves)?

- How would you, as a carer, go about finding out what they capable of doing, and what their preferences are?
- How would this knowledge affect how you look after residents – for example, if a resident had the capability, would you allow the resident to do as much for themselves as possible?

From the time I have spent here observing, I have noticed that there tends to be a ‘core’ group of residents who seem to be easier to engage in activities (be that crafty, sedentary type activities, or more active physical activities) than others.

- Does my observation sound about right to you?
- What do you do to try to persuade residents who might be less motivated to participate?
- Do you have time to do this? Do you see it as part of you job role?
- How would you go about encouraging residents to participate in any kind of activities?
- I see that you have an Activity Coordinator working here – on the days that they are here, do you do anything connected with activities for residents? What about the times when the coordinator is not here?

Some previous research has suggested that something that improves quality of life in care home residents is for them to ‘feel useful’.

- Is this something you might agree with?
- Do you ever try to get residents involved in meaningful activities (for example, housekeeping chores, assisting with mealtimes)?
- If not, why not? If you do, how do you do it, and what do you try to encourage them to do?
- How do residents interact among themselves here? Are there little groups of friends? If so is, do they ever provide support to one another?

If I were to use the term ‘physical activity’, what would you think I meant by it? *(Might ask them what their thoughts are about PA, and what they do themselves, to give a background and context)*

I am particularly interested in asking you about the amount of moving *around ( I have elected to use the term moving around rather than physical activity)* that residents do on a daily basis, and I have noticed quite a few aspects of care home residents lives across all the care homes that I have visited, that means that, on a daily basis, the residents don’t move around much.

- In the past, have you intentionally encouraged residents’ to move more?
  - How might you encourage residents to move more?
  - Think about a recent example when you encouraged someone to be physically active
  - What led you to decide to do this?
  - How did it go?
  - Anything you might have done differently?
- How do you think encouraging residents to move more fits into your job role?
- What benefits do you think moving more might bring to your residents?

During my time here, I have noticed that there are parts of the care home environment (for example, the position of tables and chairs) that might prevent or encourage residents from moving around.

- Can you tell me anything about things that you can change (quickly and easily) that might allow or encourage residents to move around a bit more?
- How much do concerns about safety (for example, a resident falling) prevent you from encouraging residents to move?
- If you see a resident starting to get up from a chair by themselves, what would be your automatic reaction? (Prompt – might you feel the need to intervene? Let them get on with it? *(Do they question a resident’s capability?)*
- How/in what ways does your organisation encourage you to support residents to be physically active (Prompt: Policy/rules; external/internal facilitators)
- Is there anything concerns or worries you that might prevent you from encouraging residents from being physically active?

Do you have any ideas about how care home residents could be more physically active? (Prompt - Any changes that could be made? Environment, social support, activities?)

- What support do you think might help, that is not already in place?
- Very generally, what sorts of things do you know about residents (in terms of their past, their preferences)
- How do you find out what might motivate a resident to be more active? How helpful do you think that information is?
- Among staff, do you discuss getting residents to move more?

Are there any initiatives that promote physical activity in care homes? How are they viewed?

*(Proxy question to try and find out what carers themselves think the purpose of care homes are)* Finally, on a personal, rather then a professional level, how would you feel about a close relative going into care home themselves?

- How would you expect them to be treated?

To finish, is there anything else you expected to talk about today, but that we did not cover?

## Topic guide for care home managers

#### Background information

Introductions, explain study, etc: I am a PhD researcher at Dundee University. Before we go on, I would like to briefly give you an overview of what this interview is about. Taking part in physical activity, such as walking, is good for health. However, we know that care home residents do not do much physical activity. To improve this, we need to understand why. One way to find out is to ask some questions about thoughts and experiences of physical activity among people who work in care homes. Whatever experiences and views you have about physical activity in the care home, we would like to hear what you think.

To begin with, would just like to ask a bit about your job here.

- How long have you worked here?
- Why did you come into this line of work?
- Enjoy job?
- Could you describe to me typical day at work?

What can you tell me about the ethos (aims, values, mission) of the service you provide here?

- In what way do you encourage staff to work to these values?
- Are there things that you think prevent your care staff from working to these values?
- Is there a culture of sharing decisions about day-to-day care between care staff and residents (for example decisions about how to spend their time)
- In what ways is staff development supported here?

What sort of environment do you aim for here? (Prompt - e.g. homelike, any particular interior design features, such as lighting, sensory stimulus such as music?)

Clearly you will work with residents relatives to a greater or lesser extent during the time that residents are with you.

- What sorts of expectations do relatives have of the service you provide?
- Are all expectations the same, or do they differ between families? How do they differ?

As a care home, you are regulated and assessed by the Care Commission

- Can I ask, how the care provided here is influenced by this regulatory oversight?

As a care home manager, you might be aware of the recently introduced Health and Social Care standards. Personally, I have scanned them, but not in depth.

- I am interested to know from your side of the fence, if you think these standards are relevant to the service you provide here, and if so how they might influence the care you provide?

I’d like to ask about normal daily life for residents in the care home, and how your residents use the care home space available to them. I have already spent quite a bit of time in the care home, simply observing residents’ day-to-day lives. During the observations that I have been doing, one of the things I have noticed is that many of the residents seem to prefer to spend time in their rooms.

- Why do you think this might be?
- As a care home, how do you balance allowing residents the choice of staying in their room with encouraging them to move?

*(Care home 2 only)* One of the other things I have noticed during my observations is that, on a daily basis, residents here only use a small amount of the available care home space. For example, whilst there are small kitchen/communal areas in each of the wings, I noticed that residents rarely use these.

- Why do you think this might be?
- Would you encourage resident to use more of the care home space that is available to them?

All residents are different, and have different abilities, but what do you believe that care home residents in general, are capable of (think about how much they can move, what decisions they can make for themselves)?

- How would you, as a carer, go about finding out what they capable of doing, and what their preferences are?
- How would this knowledge affect how you look after residents – for example, if a resident had the capability would you allow the resident to do as much for themselves as possible?

If I were to use the term ‘physical activity’, what would you think I meant by it? *(Might ask them what their thoughts are about PA, and what they do themselves, to give a background and context)*

I am particularly interested in asking you about the amount of moving around that residents do on a daily basis, and I have noticed quite a few aspects of care home residents lives across all the care homes that I have visited, that means that, on a daily basis, the residents don’t move around much. During my time here, I have noticed that there are parts of the care home environment (for example, the position of tables and chairs) that might prevent or encourage residents from moving around.

- Can you tell me anything about things that you can change (quickly and easily) that might allow or encourage residents to move around a bit more?
- How much do concerns about safety (for example, a resident falling) prevent you from encouraging residents to move?
- *(Staff)* If you see a resident starting to get up from a chair by themselves, what would be your automatic reaction? (Prompt – might you feel the need to intervene? Let them get on with it? *(Do they question a resident’s capability?)*
- How do you think encouraging residents to move more fits into your role?
- What benefits do you think moving more might bring to your residents?
- In what ways might you encourage staff to support residents to be physically active?

Do you have any ideas about how care home residents could be more physically active? (Prompt - Any changes that could be made? Environment, social support, activities?)

- What support do you think might help, that is not already in place?
- Very generally, what sorts of things do you know about residents (in terms of their past, their preferences)
- Among staff, do you discuss getting residents to move more?

Are there any initiatives that promote movement in care homes? How are they viewed?

- What features of the care home help or hinder residents to be active
  - Prompts – building layout, distances residents need to go to fulfil activities of daily living, access to outdoor space, willingness to use outdoor space, are spaces within the care home accessible (e.g. doors easily opened, door wide enough)?
- *(Proxy question to try and find out what carers themselves think the purpose of care homes are)* On a personal, rather than a professional level, how would you feel about a close relative going into care home themselves?
  - How would you expect them to be treated?
