# Supplementary material for: An Ethnographic Exploration of Social‐Ecological Influences on Physical Activity in Care Homes for Older People
Source: Health Expect. 2026 Apr 10;29(2):e70664. doi: 10.1111/hex.70664 (PMC13066766; doi:10.1111/hex.70664)
Supplement: Supplementary file 4 — Supporting File 4 [file HEX-29-e70664-s004.docx]

**Supplementary Material 4**

**Derivation of themes from the coding process**

| **Contributing codes and description** | **Themes and definition** |
| --- | --- |
| **Role development and professionalisation**  Reflects data that indicates the way in which staff member’s roles became increasingly specialised. Many carers perceived a narrow job role, with little emphasis on PA and more emphasis the provision of daily instrumental resident care.  **Operational circumstances**  Data describing the operation of the care home and any relationships and responsibilities the home has to other services and bodies – for example – links to social care provision.  **Organised exercise classes**  Data relating to formal and organised exercise sessions that reflected influences on who attends and who does not.  **Activity in relation to the everyday**  Any reference that relates to what care home residents or care home staff spend time doing. Include references to any activity other than physical activity  **Unplanned events**  Data relating to an unexpected event that disrupts a desired pattern of events  **Managing competing demands**  Instances of multiple workload demands on staff, requiring prioritisation of certain tasks over others.  **Routinisation**  Care home events that are part of the usual schedule. For example, scheduled care or activities.  **External projects**  Any reference to time-limited projects run in care homes by external agencies. In particular, a project called Care About Physical Activity (CAPA), run by the care home regulator. | **Formal role construction defines staff identity and the practice of care home work** **(Intrapersonal, interpersonal, and organisational level influences)**  This subtheme explains how staff roles were formally constructed. It defines the ways in which roles were demarcated, and how they deviated or not from a prescribed set of functions and tasks. Role construction centred around siloed professional identities and occupational boundaries that often seemed to define how care practice was enacted. Two constructs informed this subtheme:   1. *Formal organisational culture and physical activity:* values, beliefs and norms, and that led to codes of behaviour and ways of working. 2. *Identity and beliefs:* beliefs in relation to physical activity and staffs’ identity as carers |
| **Adaption of the routine**  Instances of care home staff adapting the usual daily routine of the care home in ways that facilitate physical activity  **It’s the little things that can have a big impact**  Carers emphasised that small changes that they could make, that have the potential to increase levels of care home PA  **Moving in relation to everyday activity**  Movement events that occur in relation to situational events – e.g. needing to use the toilet, or moving to dining area for a meal  **Working across boundaries**  Evidence of staff disregarding role demarcation lines and traversing boundaries to create PA opportunity  **Role values – internally driven**  Data relating to carers working outside the formal ‘rules of the game’.  **Maximising social activity**  Engagement with others (staff, other residents) on a social level, e.g. having a conversation.  **Understandings about physical activity**  Staff perceptions about physical activity, encompassing what it is, and ways it might be enacted.  **Role perception**  Any data that indicates the way in which a staff member’s role is regarded, understood or interpreted by themselves. | **Job role boundaries as blurred and informal (Intrapersonal, Interpersonal level influences)**  This subtheme explores how roles in care homes in relation to PA were - in relation to the previous subtheme - more blurred than siloed and reflected a contrast to care home staff working in discrete roles. This blurring of roles was related to the ways in which staff interacted with one another. Here, PA facilitation in relation to care home work is conceptualised as informal, in the sense that there were fewer boundaries in terms of role function among staff. This meant that roles were more fluid and that more staff were inclined see PA as within their remit. Three constructs informed this subtheme:   1. *Carers’ knowledge and understanding in relation to physical activity:* the ways in which staff knowledge and understanding of physical activity was integrated in daily care practice 2. *Carer-led informal role boundaries:* the ways in which carers interpreted their role boundaries as flexible in relation to physical activity 3. *Facilitating meaningful activity within the routine:* the ways that staff recognised scope for PA within the confines of the daily routine |
| **Espoused care home values**  Data relating to character, beliefs, fundamental values, rituals held by the care home organisation that influence behaviour and decision making in the care home. Values is the characteristic spirit of a care home which manifests in attitudes and aspirations.  **Enabling versus dependency**  Data indicating tension between enabling (allowing residents to ‘do’ for themselves) and then ‘doing for’ residents which might foster dependency.  **Perceptions of capability**  The way in which residents, their family and care home staff perceive residents’ capacity for movement and participation in physical activity | **Tension between task focused care, espoused values, and physical activity (Intrapersonal, organisational level influences)**  This subtheme captures the dynamics of divergence between work practices driven by espoused values and actual performances. It explores how rigid care home routines that were intended to ensure that tasks related to basic/functional care (for example, getting residents up and dressed, meal provision and medication administration) and tasks related to broader care home operation (for example administrative duties and staff meetings) led to staff disempowerment for PA by being at odds with care homes espoused values and PA promotion. This subtheme was informed by two constructs:   1. *Espoused values and task-focused care versus actual care practices* 2. *Resisting disempowerment and circumventing the rules* |
| **Role values – expectations**  Drivers of the work of carers which are informed by perceived external expectations (for example from family members and social work professionals) of the role. This informed how the carer’s role was regarded, understood and interpreted by themselves.  **Staff versus management perceptions**  Differing views between what carers see as PA and what management and senior staff see as PA. | **Tension in expectations (Interpersonal level influences)**  This theme reflects tensions related to expectations of care practices that led to disempowerment of carers from facilitating PA. Rather than each care home’s own espoused values as drivers of care practice (chapter six), what actually appeared to influence the nature of PA promotion in care homes were expectations of the nature of care that emphasised a more traditional approach of ‘doing things for’ residents rather than allowing residents to do as much as possible for themselves in a way that facilitated PA. These expectations were articulated by residents, families, and to some extent external organisations such as social work. |

| **Contributing codes and description** | **Subthemes and description** | **Final main theme and description** |
| --- | --- | --- |
| **Situational influences on movement**  Movement that relates to the location and surroundings of a place. Therefore, physical activity or movement relates to, or is dependent upon a set of circumstances.  **Physical infrastructure – fixed**  Aspects of the physical environment that could influence physical activity that are fixed an immovable. For example, doorways, halls, steps.  **Use of space - care home**  Any data relating to how residents use the available care home space.  **Ambient environment**  Data that relates to environmental factors beyond the build and physical environment such as weather and background care home noise.  **Resources**  Assets that staff draw upon to facilitate physical activity, such knowledge of residents’ interests.  **Habitual movement**  Instances where care home residents move out of habit. Includes wandering behaviours. | **Spatial dynamics**  This subtheme explores the differences between physical spaces that made them less or more likely to be used in dynamic ways to meet individual resident, as well as collective needs. Spatial dynamics includes various environmental aspects such as sounds, view, layout, and privacy, all of which coalesced to denote the purpose of different spaces in the homes. Coupled with the theme *Staff empowerment and disempowerment from promotion of physical activity* (explored in chapter six), this subtheme explores how aspects of the physical environment created complex situations that existed between care, creating PA opportunity, and control. This subtheme is discussed through three concepts:   1. *Destinations as incentives for physical activity* 2. *Communal spaces* – *autonomy and opportunity* 3. *Encouragement from staff* | **Social spaces as destinations for physical activity**  Social spaces refers to physical spaces in care homes to which all residents have access, and where residents were observed and reported to congregate. This theme captures the notion of different physical areas of care homes emerging as social spaces and in turn, ‘destinations’ for residents to walk to, and explores the use of these areas (and, crucially, getting to these social spaces) as an important manifestation of PA. Here, Social spaces facilitated PA by providing access to appropriate resources, support, and (re)enforcement of group or social norms for PA promotion, and formed destinations that led to the emergence of informal ‘paths’ for residents to walk within the care home, thus facilitating residents’ PA.  The theme reflects how, in tandem with the social environment explored in chapter six, the physical environment shaped: (1) resident’s use of the available care home space; (2) how residents appropriated available care home space as destinations, and (3) the extent of social support as related to physical activity afforded to residents by these social spaces.  Social spaces as destinations for PA were determined by the nature of *spatial access,* the extent of *spatial control*, and *spatial dynamics,* all of which led to the forming of social spaces as destinations for walking. |
| **Accessing external areas**  Data relating to how residents access external areas such as gardens.  **Security**  Data relating to the ways in which security features (such as locked doors) influenced resident movement around the care home. | **Spatial access**  Captures the ease with which residents in a given area of the care home were able to access different areas of the care home. Layout and wayfinding (for example, environmental adaptations such as signage) emerged as important influences. Two concepts informed this subtheme:   1. *Barriers to accessing social spaces* 2. *Access to social spaces* |  |
| **Physical infrastructure – adaptable**  Aspects of the physical environment that are easily amenable to changing or altering by care home staff. For example, moving furniture, changing room use.  **Situational influences on movement**  Physical environmental features initiated by staff to encourage resident movement  **Flexible approach**  Data relating to staff demonstrating a flexible approach to their role. | Main theme only | **Environmental adaptation**  This theme explores the ways in which staff felt able and empowered to respond to: (1) perceived inadequacies and, (2) the potential for manipulation of the physical environment in ways that related to physical activity. The theme captures staff’s perceptions and reflections on the role of the physical environment in PA, and how staff were best placed to identify opportunities for optimising physical environment pathways for PA through facilitating resident autonomy and personal control.  Staff appeared to enact strategies in response to perceived physical environmental needs in two ways. First at the scale of the spatial organisation of the care home to create a variety of destinations as social spaces for various activities and social interactions, both indoors and outdoors, and secondly at the scale of the design and layout of specific spaces.  The theme, therefore, emphasises the use of space in care home, as well as staff capacity to create an enabling physical environment that was conducive to PA. The theme reflects how staff actively made use of the existing inherent possibilities of the physical environment in response to a perceived need for residents to be physically active and explores how staff identified flexible aspects of the physical environment that were amenable to manipulation. Importantly, the premise of this theme is that adaptations made to the physical environment are contingent on the empowerment, flexibility and creativity of staff (chapter six). |
